# Mechanical Properties of PC-ABS-Based Graphene-Reinforced Polymer Nanocomposites Fabricated by FDM Process

**DOI:** 10.3390/polym13172951

**Published:** 2021-08-31

**Authors:** Vijay Tambrallimath, R. Keshavamurthy, Saravana D. Bavan, Arun Y. Patil, T. M. Yunus Khan, Irfan Anjum Badruddin, Sarfaraz Kamangar

**Affiliations:** 1 Department of Automobile Engineering, Dayananda Sagar College of Engineering, Bangalore 560078, India; vijay-au@dayanandasagar.edu or; 2 Department of Mechanical Engineering, Dayananda Sagar College of Engineering, Bangalore 560078, India; 3 Department of Mechanical Engineering, Dayananda Sagar University, Bangalore 560078, India; saranbav-me@dsu.edu.in; 4 School of Mechanical Engineering, KLE Technological University, Hubballi 580031, India; arun_p@kletech.ac.in or; 5 Department of Mechanical Engineering, College of Engineering, King Khalid University, Abha 61421, Saudi Arabia; yunus.tatagar@gmail.com (T.M.Y.K.); magami.irfan@gmail.com (I.A.B.); sarfaraz.kamangar@gmail.com (S.K.)

**Keywords:** polymer nanocomposite, FDM, graphene, mechanical properties

## Abstract

This experimental study investigates the mechanical properties of polymer matrix composites containing nanofiller developed by fused deposition modelling (FDM). A novel polymer nanocomposite was developed by amalgamating polycarbonate-acrylonitrile butadiene styrene (PC-ABS) by blending with graphene nanoparticles in the following proportions: 0.2, 0.4, 0.6, and 0.8 wt %. The composite filaments were developed using a twin-screw extrusion method. The mechanical properties such as tensile strength, low-velocity impact strength, and surface roughness of pure PC-ABS and PC-ABS + graphene were compared. It was observed that with the addition of graphene, tensile strength and impact strength improved, and a reduction in surface roughness was observed along the build direction. These properties were analyzed to understand the dispersion of graphene in the PC-ABS matrix and its effects on the parameters of the study. With the 0.8 wt % addition of graphene to PC-ABS, the tensile strength increased by 57%, and the impact resistance increased by 87%. A reduction in surface roughness was noted for every incremental addition of graphene to PC-ABS. The highest decrement was seen for the 0.8 wt % addition of graphene reinforcement that amounted to 40% compared to PC-ABS.

## 1. Introduction

Thermoplastics and thermoset plastics are two categories of polymer materials that are used based on application. Thermoplastics polymers are the most abundantly available materials that are used in the FDM process due to lower cost and lower melting temperature [1]. The most commonly used thermoplastic polymers are PLA, ABS, and nylon, used in FDM [2,3]. There is a specific constraint with the use of these polymers alone in achieving the desired properties. As a result, it is critical to improve fabricated parts’ properties, which could be accomplished by adding filler material. Polymer alone would not provide the required mechanical properties; thus, filler material in macroparticles, microparticles, or even nanoparticles is used to improve the properties. Several researchers have experimented with various filler material and matrix combinations in order to strengthen specified properties.

Three-dimensional printing, also known as additive manufacturing (AM), is gaining popularity among industrialists and researchers. According to Ford et al., fused deposition modeling (FDM), a significant shareholder of AM in the current market, uses polymer in the form of filament as a raw material to develop either prototype or functional models. Any complex model can be quickly created using FDM. The printed model is created in any CAD format and then converted to STL file format, which is then sliced and fed into the 3D printer. The option of using AM opens up a wide range of possibilities for improving design properties and printing speed [2]. Keshavamurthy et al. have discussed green manufacturing technology and the feasibility of developing any intricate part through additive manufacturing using various methods. Additive manufacturing is classified into seven categories based on the material and application [3]. Kazmer et al. have investigated a wide range of polymer properties. Plastics have been widely used in automotive, aerospace, dentistry, electronics, and medicine due to their lightweight quality, manufacturing stability, processability, and low cost. These benefits have paved the way for the use of plastics in FDM [4].

Melenka et al. conducted an experimental study with a polymer matrix as ABS and a reinforcement of Kevlar fibers. To test the tensile properties, the composite was created with different volume percents of 4.04 percent, 8.08 percent, and 10.1 percent. It was discovered that increasing the filler content increased the Young’s modulus and tensile strength [5]. Perez et al. conducted a comparative study of tensile strength variation with various filler materials. ABS was used as a matrix material to create the samples. TiO2, jute fiber, and thermoplastic elastomer (TPE) were added separately compared to the pure ABS sample. Tensile strength increased for ABS-TiO_2_ compared to pure ABS; however, tensile strength decreased for jute fiber and TPE [6]. Vijay et al. investigated thermal conductivity with the addition of Cu nanoparticles. The shape of the nanoparticle would greatly influence the desired properties. Cu was added in the proportions of 2.5 wt % and 5 wt %, respectively. With the addition of 5 wt percent Cu, the value was increased. The findings were consistent with the mathematical models [7].

Brennan et al. investigated the improvement of mechanical properties for low loading of graphene oxide with carboxyl and hydroxyl functional groups. The addition of nanofiller material would significantly improve the required properties for FDM-printed parts. The improvement in the multifunctional property was observed for the addition of less than 0.1 wt percent of GO [8]. Lin et al. conducted an exploratory study to improve mechanical, thermal, and electrical properties with appropriate filler content developed by the 3D printing process. However, the addition of nanomaterials would enhance one property while compromising another [9]. The addition of 10% carbon nanotubes increased tensile strength by 7.5 percent, but there was a decrease in elongation to failure and an increase in brittleness, according to Sandoval Jr. H. et al. [10]. Wei X. et al. proposed that adding 5.6 wt percent graphene to ABS would increase the electrical conductivity of polymer nanocomposite by four orders of magnitude [11]. The addition of TiO_2_ could improve thermal stability, and Weng Z. et al. [12] investigated nanoclay.

Vijay T. et al. recently investigated the filament characterization of a PC-ABS filament reinforced with graphene nanofiller. The filler was added in increments of 0.2, 0.4, 0.6, and 0.8 weight percent. The SEM and elemental mapping provided useful information about the dispersion of filler content in filaments produced by the twin-screw extrusion process. The presence of filler had no effect on the diameter of the extrusion wire [13]. The developed filament was limited to the analysis of dispersion characteristics in this work, and no studies on the fabrication of 3D-printed parts were conducted. The current paper delves into 3D printing of parts and the determination of their mechanical properties and surface roughness, allowing for a better understanding of the impact of filler addition and its use in engineering applications.

Certain polymers have distinct properties and serve as a tough material. Polycarbonate (PC) is one such polymer with a high toughness and resistance value. It can behave as a brittle material under certain strain conditions. The addition of a limited amount of ABS to PC resulted in balancing certain properties and improvements in mechanical stability and economic value [14]. Polymer fusion of PC and ABS yields a new class of materials with improved strength and processing properties. Because the combination of these two polymers results in more flexible processing characteristics than ABS and greater strength than PC, it is the material of choice for fused deposition modelling (FDM) [15,16]. The material’s properties can be improved by the addition of macro- or nanosized particles. Because of their superior surface-to-volume ratio and ability to form network chains with polymer matrix and disperse in a homogeneous manner, nanoparticles outperform macroparticles [17]. The addition of nanoscale reinforcement to the polymer matrix results in the use of polymer nanocomposites in a wide range of engineering sectors, including automotive, aerospace, construction, and packaging. Graphene has a 2D lattice structure with very high thermal and electrical conductivity and enhanced mechanical properties, making it a highly sought after material. The properties of graphene have led to its use in metal, ceramic, and polymer matrix materials [18,19]. Industries such as electronics, green energy, aerospace, and automotive have made extensive use of polymer matrix nanocomposites, particularly graphene as reinforcement. As previously stated, graphene is a two-dimensional material with good electrical, mechanical, and thermal properties and a higher aspect ratio and surface area than other forms of reinforcement such as CNTs, carbon fibers, and so on. The addition of graphene as reinforcement would result in a significant improvement in engineering properties. With an extensive literature review, the major research gaps identified were in the development of nanocomposite filaments, the study of desired engineering properties, and the lack of optimal process parameters. To elaborate, firstly, it was difficult to achieve desired physical, mechanical, and thermal properties in 3D-printed polymer products, resulting in limited engineering applications. Second, only a few studies on the use of graphene as reinforcement in polymer matrix development via FDM were conducted. Third, there is still a significant gap in mechanical studies of polymer nanocomposites in the development of thin-layer sections with high-performance engineering applications. Finally, the combination of PC-ABS as a polymer matrix was used in a negligible amount.

The extraction of PC-ABS in the form of filament, which is required for FDM, could be performed in a smooth flow while retaining the desired properties. To create the composite, graphene, a novel material with extremely high mechanical and thermal properties, is used as nanofiller. In the current study, graphene is used as a filler material in the PC-ABS matrix at 0.2, 0.4, 0.6, and 0.8 wt percent. Physical and mechanical properties of the developed polymer composite are tested.

## 2. Materials and Methods

**Figure 1:**
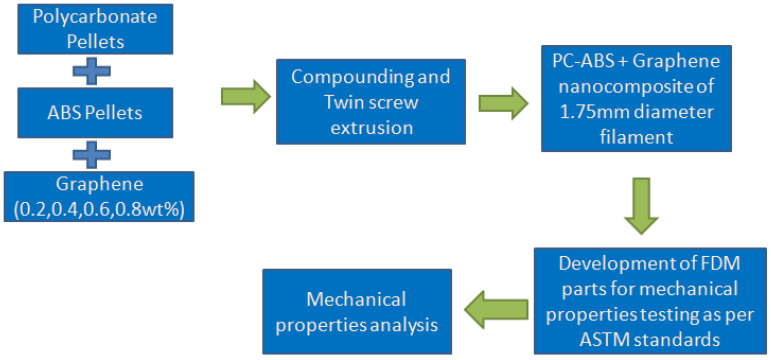
Flowchart of process flow.

The following Figure 1 represents the flowchart that describes the process flow of experimental study.

### 2.1. Nanocomposite Preparation

**Figure 2:**
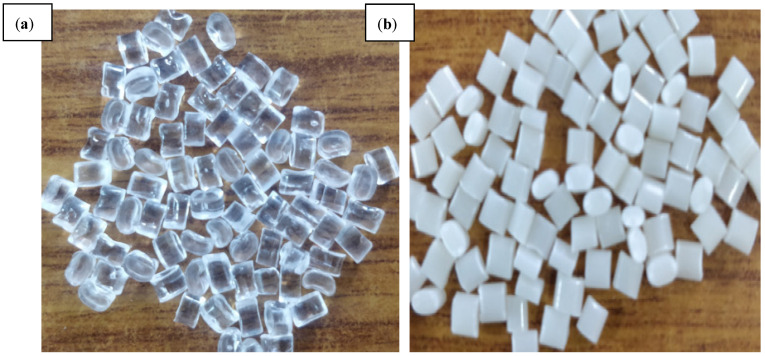
Photographs of (**a**) PC and (**b**) ABS.

**Figure 3:**
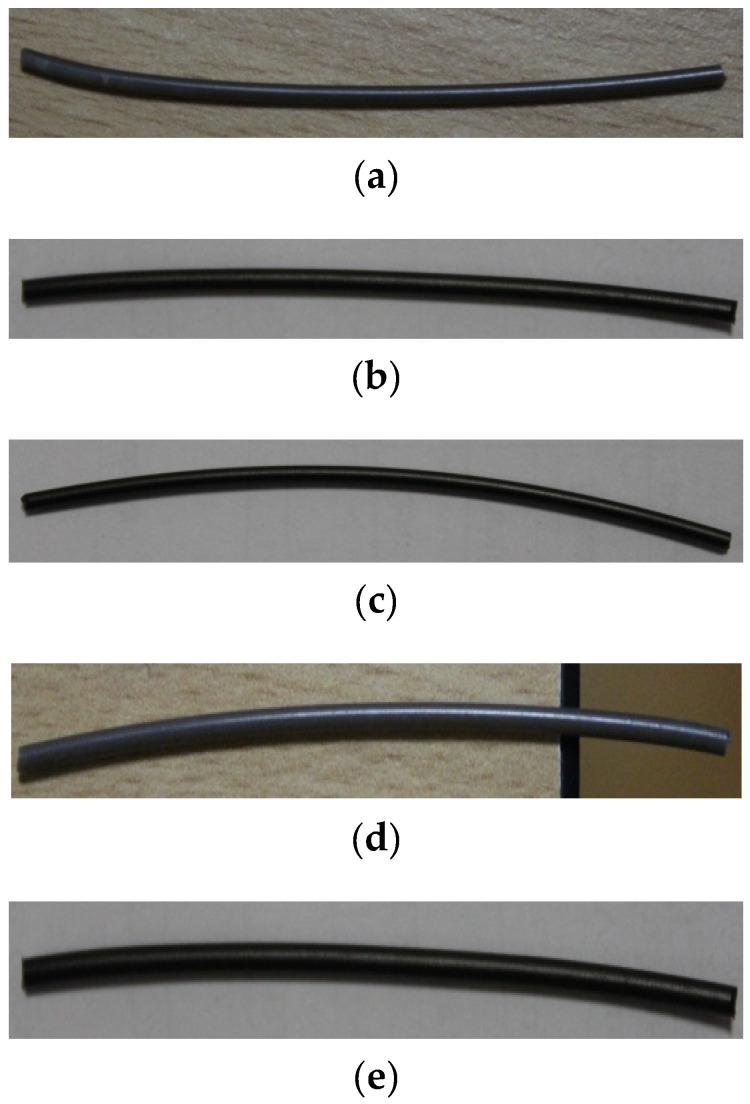
Photograph of filaments with incremental filler content. (**a**) PC-ABS. (**b**) PC-ABS + 0.2 wt % graphene. (**c**) PC-ABS + 0.4 wt % graphene. (**d**) PC-ABS + 0.6 wt % graphene. (**e**) PC-ABS + 0.8 wt % graphene.

PC and ABS in the form of pellets were mixed in the ratio 70:30. The parts to be fabricated by polymers were obtained in the form of pellets. As shown in Figure 2, PC and ABS pellets were separately procured. Drying plays an essential role in removing moisture and making the flow process smooth; hence, at a temperature of 120 °C the pellets were dried for 4 h. Once the process of drying was completed, these dried pellets were added to the process of compounding. Graphene in the form of multilayers with uneven shape was added as reinforcement in definite measured quantities to these dried pellets and was extracted out as a filament. These extracted filaments were cut into pellets and dried at 100 °C and were later fed into the extrusion machine. A lab grade compounding machine was used and a single screw double rod extruder was used for extrusion of the filament of 1.75 mm diameter. Figure 3 shows the short length of filaments developed through the twin-screw extrusion process used for specimen development.

The addition of graphene has exhibited proper amalgamation with matrix material, leading to even surface diameter of the filament. The macroanalysis showed no cracks or flaws in the developed filament, and microanalysis did not exhibit any visible defects. The addition of graphene reinforcement to the matrix showed the diameter readings to be similar to that of pure PC-ABS without any effect on surface smoothness.

### 2.2. Microstructure Analysis

JSM 7100F Jeol model field emission scanning electron microscope was used for microstructure analysis of graphene. Scanning electron microscope (JSM 840a Jeol, Bangalore, India) was used for SEM and EDAX studies.

### 2.3. XRD

Philips X’Pert Pro X-ray diffractometer was used for taking XRD patterns on developed composites. This ray is diffracted from the specimen and is recorded in the acquisition software.

### 2.4. Raman Spectroscopy

The Raman spectrum was recorded with Peak SeekerPro^TM^ Raman system, Bangalore, India. First, a 785 nm wavelength laser with a power of 5–300 mW was implemented in excitation of the sample. A 100 micron laser spot size was noted. The system comprises TE cooled, efficient CCD detector arrays which are cooled at −20 °C. A vial holder is used to place the sample. High sensitivity, resolution, and stability of the machine are met with utilization of USP monograph 1120. Rayleigh scatters are filtered by deep blocking laser, which helps isolate Raman scatter for valuable molecular analysis. The Raman spectra were recorded with RSIQ software. The resolution of the spectra is ~6 cm^−1^. The accumulation time is 5 s.

### 2.5. Fused Deposition Modelling (FDM)

**Figure 4:**
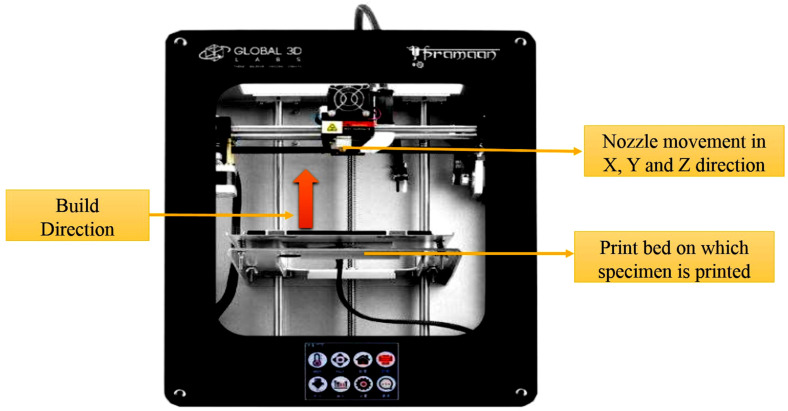
Photograph of FDM machine used for fabrication of parts.

FDM follows a process of developing the models by the addition of material layer-wise. FDM is one of the most widely used 3D manufacturing techniques used for the development of polymer parts. The 3D model is developed through a CAD file. Any complex part can be easily developed through this process [19]. A pramaan printer from Global 3D labs, Bangalore, India, was used to develop tensile and impact test specimens. A 4000 mm^3^ enclosed chamber was the build volume of the printer. Optimal parameters were chosen for the development of models as follows: infill density of 100%, layer thickness of 0.1 mm, shell thickness of 0.4 mm, top and bottom layer thickness of 1.2 mm, speed of 5 mm/s, the orientation of 45°, the temperature of the nozzle for PC-ABS fabrication maintained at 240 °C and varied for other proportions, and bed temperature maintained at 80 °C. The 3D printer used for printing is shown in Figure 4. The nozzle traces its path in X, Y, and Z direction through which the filament is passed and printed on the print bed. The parts are fabricated in Y direction orientation.

### 2.6. Surface Roughness

**Figure 5:**
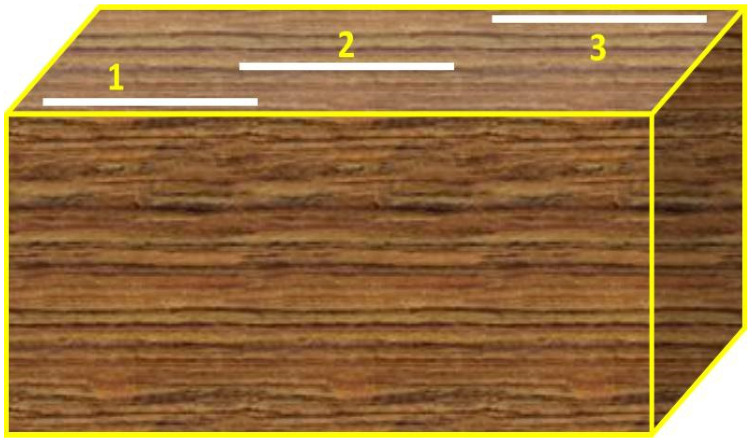
Measurement of surface roughness on the top surface at various positions.

Surface roughness (Ra) was measured for the FDM parts using Miyu surface roughness tester (M35:2010), Bangalore, India. The specimen of 10 mm × 10 mm × 20 mm was developed for measurement of surface roughness Ra, through which three trials were conducted at various lengths of the surface as shown in Figure 5 and the average value of the roughness was analyzed. Ra represents the arithmetic mean deviation of the concerned profile.

### 2.7. Tensile Test

**Figure 6:**
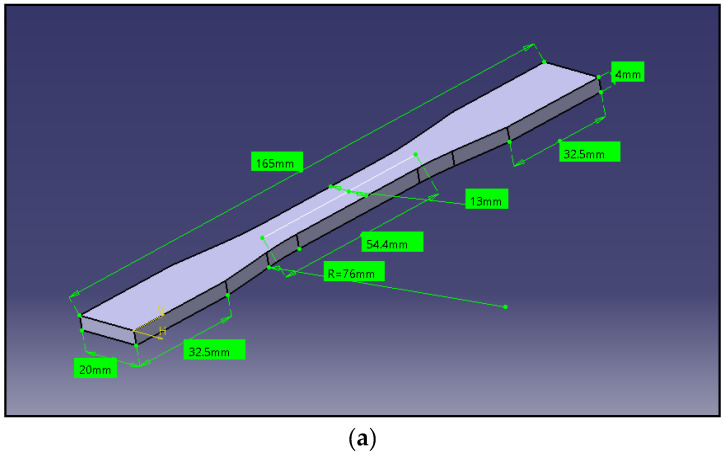

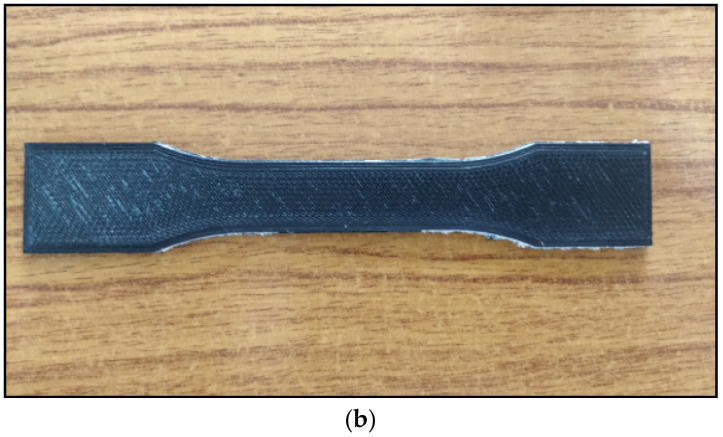
(**a**) Tensile specimen dimensions. (**b**) Photograph of tensile specimen.

ASTM D638 standard procedure was implemented for conduction of tensile test [20], make of Fuel Instruments and Engineers Pvt. Ltd. (FIE), Bangalore, India, a machine with a capacity of 0–60 tons was used. The tensile test was carried out at a speed of 2 mm/m at ambient temperature. Each tensile strength reading for various compositions of composites was taken as an average of eight specimens. Figure 6 shows the dog bone model ASTM dimensions and a photograph of the tensile test specimen. The study was conducted to identify the tensile properties with an increase in graphene content.

### 2.8. Impact Tests

**Figure 7:**
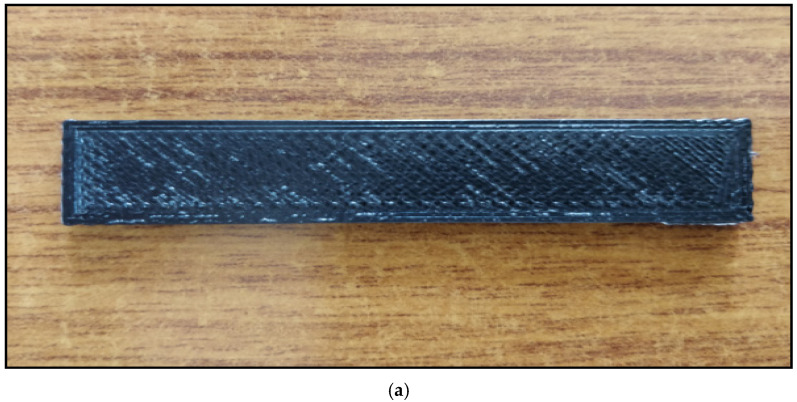

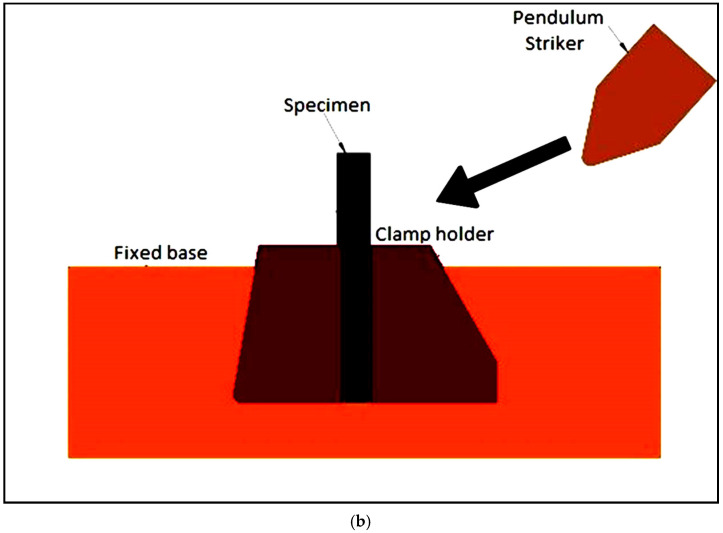
(**a**) Photograph of impact test specimen developed by FDM. (**b**) Photograph of scheme of loading during Izod impact test.

FDM parts’ ability to hold the load with the increase in filler content was analyzed using Izod impact test following a procedure of ASTM D4812 [20]. The test was carried out with equipment make of Fuel Instruments and Engineers Pvt. Ltd. (FIE), Bangalore, India, with a capacity of 0–60 tons. The specimen developed for impact test using FDM is shown in Figure 7a,b shows the scheme of loading during the Izod impact test. The maximum failure energy corresponding to the hammer of the pendulum is 300 + 10 J; the speed of the pendulum at impact time is 5 m/s. Low-velocity impact test was carried out to analyze the impact resistance ability of the developed part for its utilization in various specified sectors. The impact strength has been calculated as per Equation (1).

K = W/A(1)

where K—Impact Strength, W—Impact Energy recorded on scale, and A—Area of the specimen.

## 3. Results and Discussion

### 3.1. X-ray Diffraction Analysis and Raman Spectroscopy of Graphene

**Figure 8:**
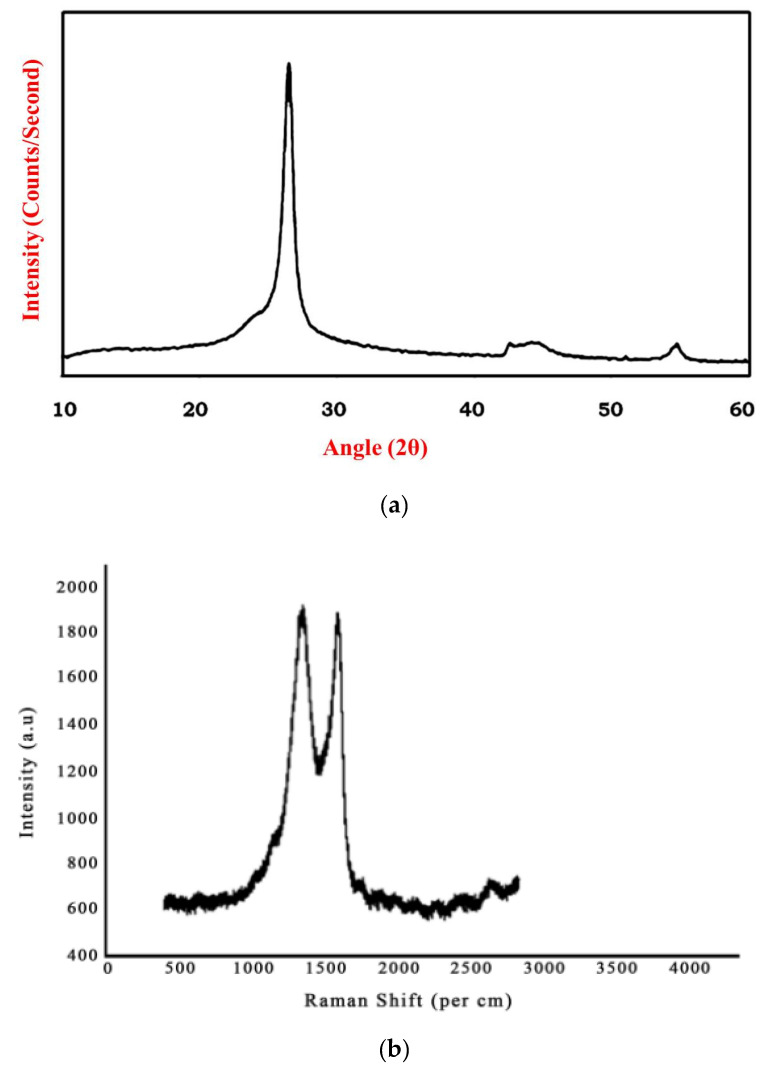
(**a**) XRD analysis graphene. (**b**) Raman spectrograph of graphene.

The obtained XRD pattern for graphene is represented in Figure 7. Pristine graphene exhibits a basal reflection (002) sharp peak at 2θ = 27.0° corresponding to a d spacing of 3.370 Å in graphite layer structure. The high intense peak shows the crystalline nature of graphene. Figure 8a shows the XRD analysis of graphene. Figure 8b shows the Raman spectrum for graphene. The presence of conjugated and carbon–carbon double bonds leads to the formation of high-intensity peaks. The G band of the graphene is seen to occur at approximately 1580 cm^−1^. The D band indicates the presence of disorder either in vacancies, grain boundaries, and carbon content’s amorphous nature. Johra et al. [21] obtained similar Raman spectra in their study on graphene preparation.

### 3.2. Surface Morphology Analysis

**Figure 9:**
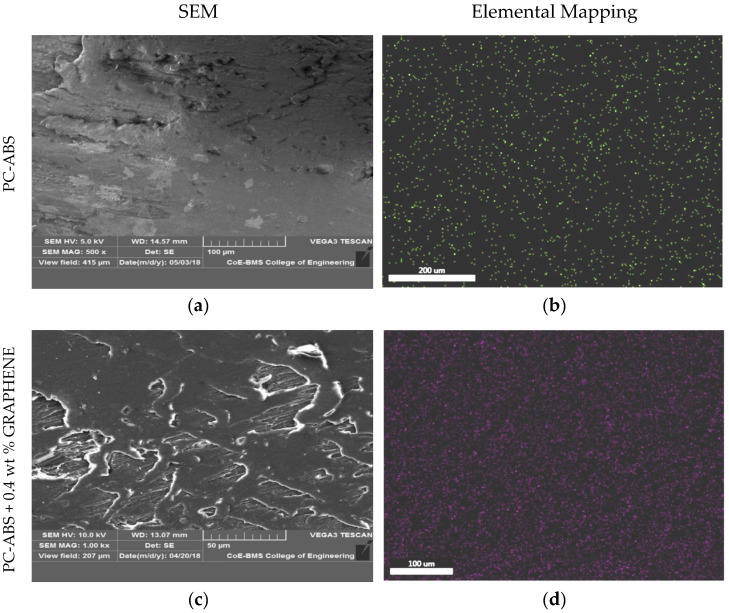

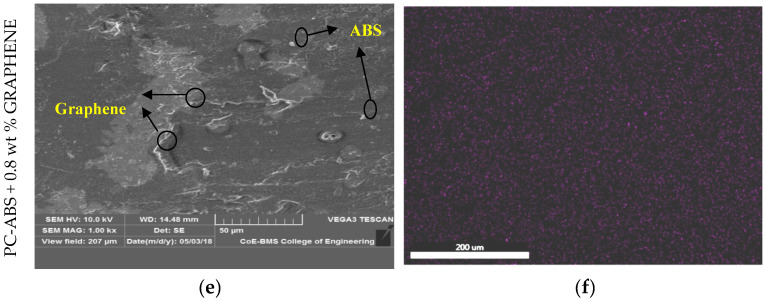
SEM and elemental mapping of (**a**,**b**) PC-ABS, (**c**,**d**) PC-ABS + 0.4 wt % graphene, and (**e**,**f**) PC-ABS + 0.8 wt % graphene.

**Figure 10:**
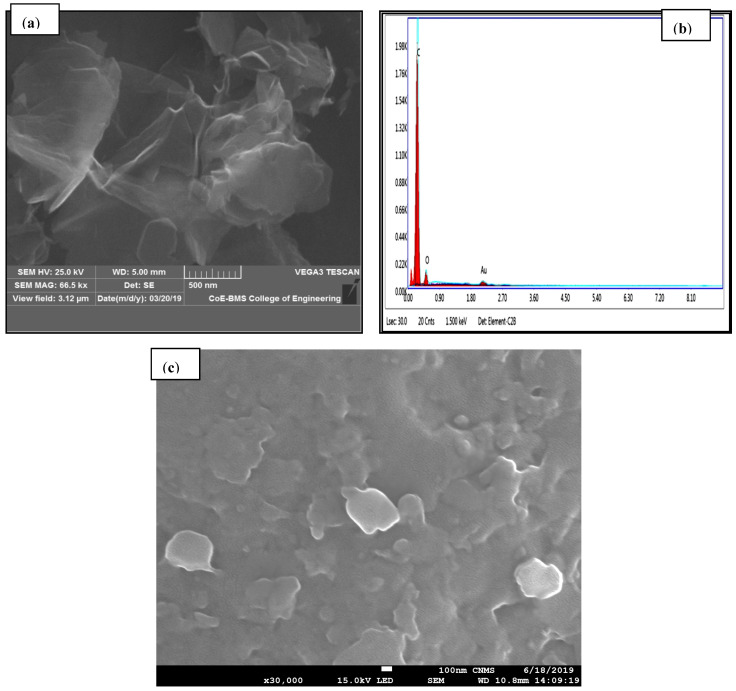
(**a**) SEM of graphene-reinforced PC-ABS. (**b**) EDAX of graphene. (**c**) Graphene dispersion in PC-ABS.

Figure 9 depicts PC-ABS’s microstructure and elemental mapping and its composites, indicating the dispersion of filler material in the matrix without agglomeration. Preheating, extrusion temperature, and compounding temperature are important factors in forming well-dispersed nanocomposite filament. It was also discovered that increasing the filler content improves the dispersion properties of the filler, allowing for an increase in mechanical properties. Utilization of developed filaments in FDM did not lead towards clogging of the nozzle [13]. Figure 10 depicts an SEM image of graphene with EDAX (a,b). The dispersion of graphene nanoparticles in the PC-ABS polymer matrix is investigated, revealing the filler material’s homogeneous distribution without agglomeration. The elemental mapping images show no visible aggregation of graphene reinforcement, indicating better properties with a 2D lattice structure and visibly quantifying a better surface-to-volume ratio. Graphene has been evenly distributed throughout the matrix. The reinforcement has an irregular shape.

**Figure 11:**
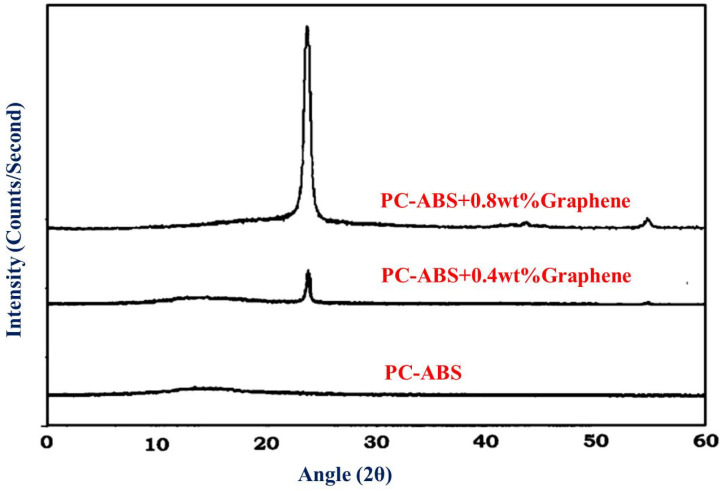
XRD of PC-ABS, PC-ABS + 0.4 wt % graphene, and PC-ABS + 0.8 wt % graphene.

Figure 11 depicts ABS dispersion in spherical form in the PC matrix and uniformly dispersed graphene in an irregular shape with no debonding formation. Pour et al. [14] obtained similar results regarding the distribution of ABS in PC, with addition of ABS ranging from 0 to 40 wt % in PC resulting in dispersion in the form of nodules or fibrils [22]. The efficacy involved in interfacial interaction and dispersion is depicted by the matrix’s lower or higher addition of reinforcement content. This dispersion property allows transfer from polymer to nanofiller, increasing mechanical and thermal properties over pure polymer. The presence of a functional group in graphene allows interaction with PC-ABS; additionally, the lower concentration of graphene suffices to provide the required dispersion characteristics. The addition of a greater amount of graphene causes interaction between their van der Waals forces and the formation of agglomeration. Because of the presence of van der Waals forces, graphene layers constantly tend to agglomerate. In practical considerations, standalone graphene could not exist because of thermal variations; as well, the steadiness of the long-range crystalline order found in graphene was thought to be impossible at room temperature. Graphene’s individual layers undergo scrolling, crumpling, folding, and wrapping, making it suitable for improving the performance of polymers [23].

The studies of the dispersion of nanofiller have stated that graphene dispersion occurs in the styrene-acryolnitrile phase and not in the polybutadiene phase of ABS [24]. Gao et al. [25], have also reported the dispersion of graphene in styrene-acrylonitrile phase, due to which the strong π–π interactions happen with phenyl rings of styrene-acrylonitrile and graphene.

### 3.3. X-ray Diffraction (XRD)

Figure 11 shows the XRD patterns of pure PC-ABS and PC-ABS reinforced with graphene at 0.4 and 0.8 wt %. PC-ABS does not display any distinct peaks indicating the matrix to be in amorphous nature. The presence of graphene indicates a sharp peak at 2θ = 26.5°. The corresponding 2θ angle relates to the plane (002) for graphene [26,27], with recorded *d* spacing of 3.36 Ᾰ. Thus, the presence of peaks proves the existence of graphene in the PC-ABS matrix, with multilayer graphene having a specified *d* spacing. The FESEM image shown above in Figure 9, Figure 10 and Figure 11 substantiates the homogenous dispersion of graphene.

**Figure 12:**
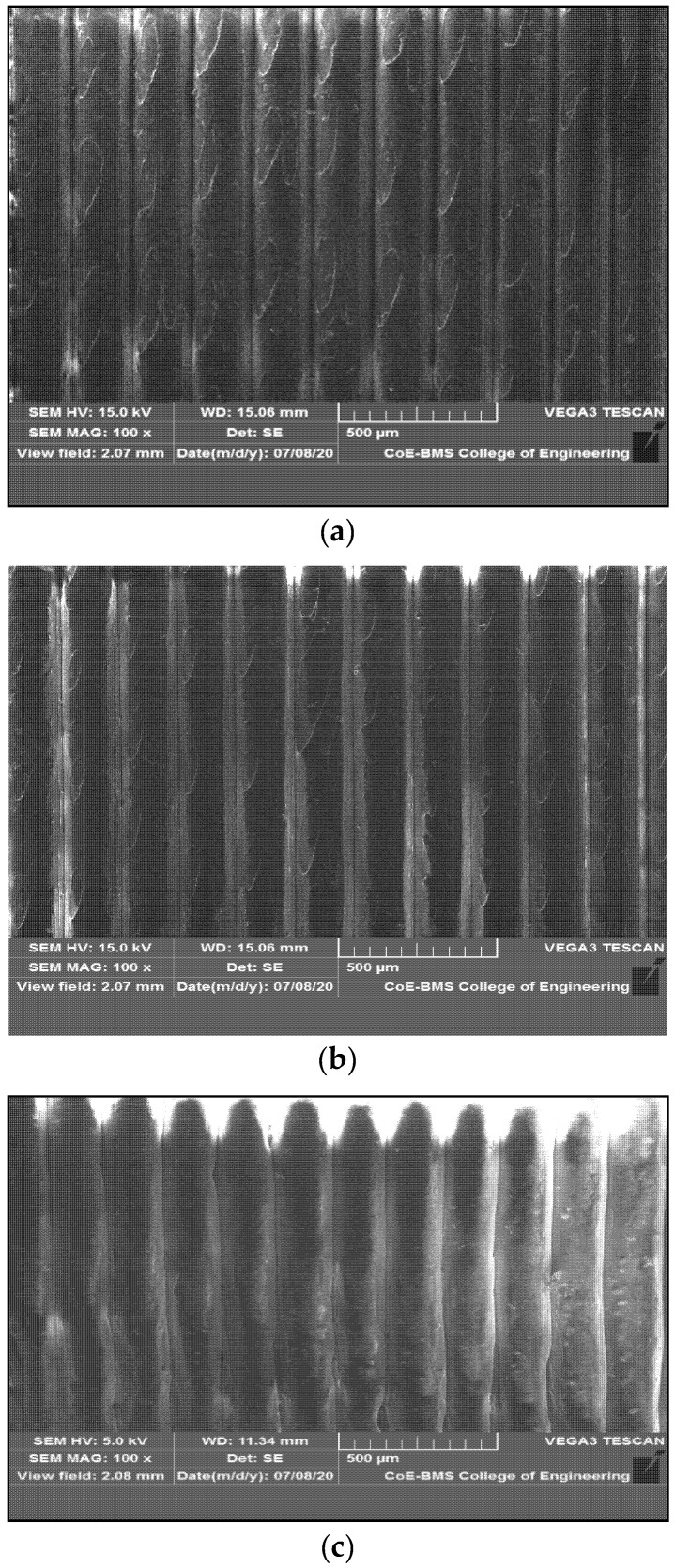

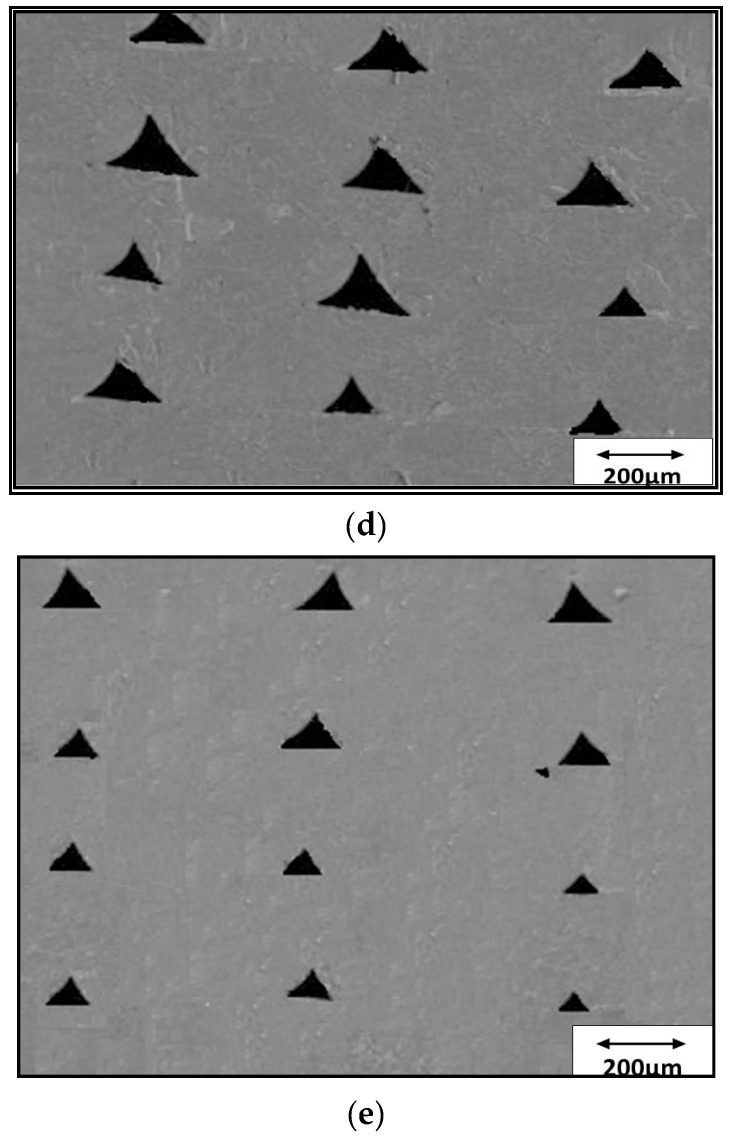
(**a**) SEM of vertical plane section of FDM-printed surface of PC-ABS. (**b**) SEM of vertical plane section of FDM-printed surface of PC-ABS + 0.4 wt % graphene. (**c**) SEM of vertical plane section of FDM-printed surface of PC-ABS + 0.8 wt % graphene. (**d**) SEM of void formation in PC-ABS. (**e**) SEM of void formation in PC-ABS + 0.8 wt % graphene.

The first layer of filament that is laid on the heating bed from the nozzle forms a binding zone between the adjacent filament surfaces that are laid one above the other; gradually, the diffusion occurs because of variation in temperature between the adjacent layers and also between the layer and the bed, the rapid cooling of the layers leading in enhancing the bonding between the layers [28,29]. However, there remains the void between the filaments, which takes the shape of a triangle rather than a circle. The enhanced polymer diffusion with graphene content reduces pore size, leading to stronger bond formation. However, the formation of voids would not be possible between layers when the fill density is 100%; hence, there is no notable void in the images seen in Figure 12a–c for PC-ABS, PC-ABS + 0.4 wt % graphene, and PC-ABS + 0.8 wt % graphene. The formation of porosity is seen in PC-ABS in Figure 12d, with the appearance of triangular spaces at the juncture of connection between two layers. Figure 12e shows the formation of the similar pores for PC-ABS + 0.8 wt % graphene addition; however, the decrement in pore sizes has been noted with the increased addition of graphene. Wang et al. reported a similar observation in [30], suggesting that increased temperature of nozzle and build platform will lead to diffusion and enhancement of bond strength.

### 3.4. Surface Roughness

**Figure 13:**
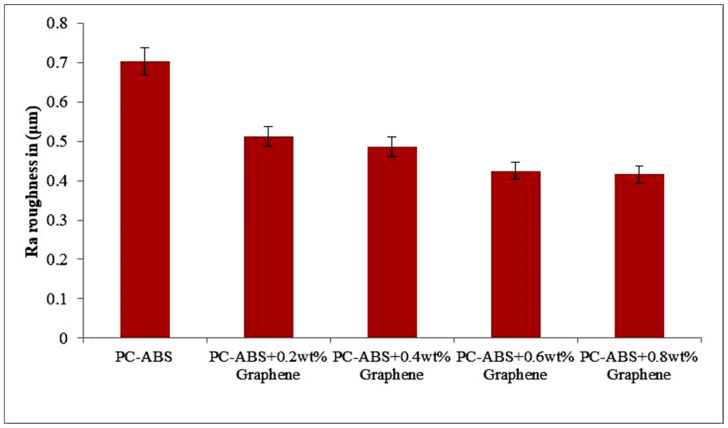
Variation of surface roughness with the addition of graphene in X direction (build direction).

The roughness (Ra) average values are used to compare the roughness with different specimens and are plotted in the graph as shown in Figure 13. The average surface roughness values are presented in Table 1. The graph indicates the decrease in surface roughness with the addition of graphene content. The increase in surface finish value may be attributed to dimensional stability and homogenous dispersion of the reinforcement. Homogenous distribution helps in the enhancement of thermal conductivity and also improvising dimensional stability [31].

Compared to pure PC-ABS addition of 0.2 wt % of graphene, it has a decrement of 25.8% in surface roughness. Similarly, the decrement trend is followed with addition of reinforcement. The addition of 0.4 wt % graphene PC-ABS saw a reduction of 11.1% in comparison to 0.2 wt % graphene addition. Similarly, a reduction of 12.5% was noted for 0.6 wt % addition of graphene compared to 0.4 wt % addition. The highest decrement is seen for the 0.8 wt % addition of graphene reinforcement that amounts to 40% compared to PC-ABS. The calculation of error bars was performed by considering the standard deviation and dividing it by square root of the overall measurements that constitute the mean.

**Figure 14:**
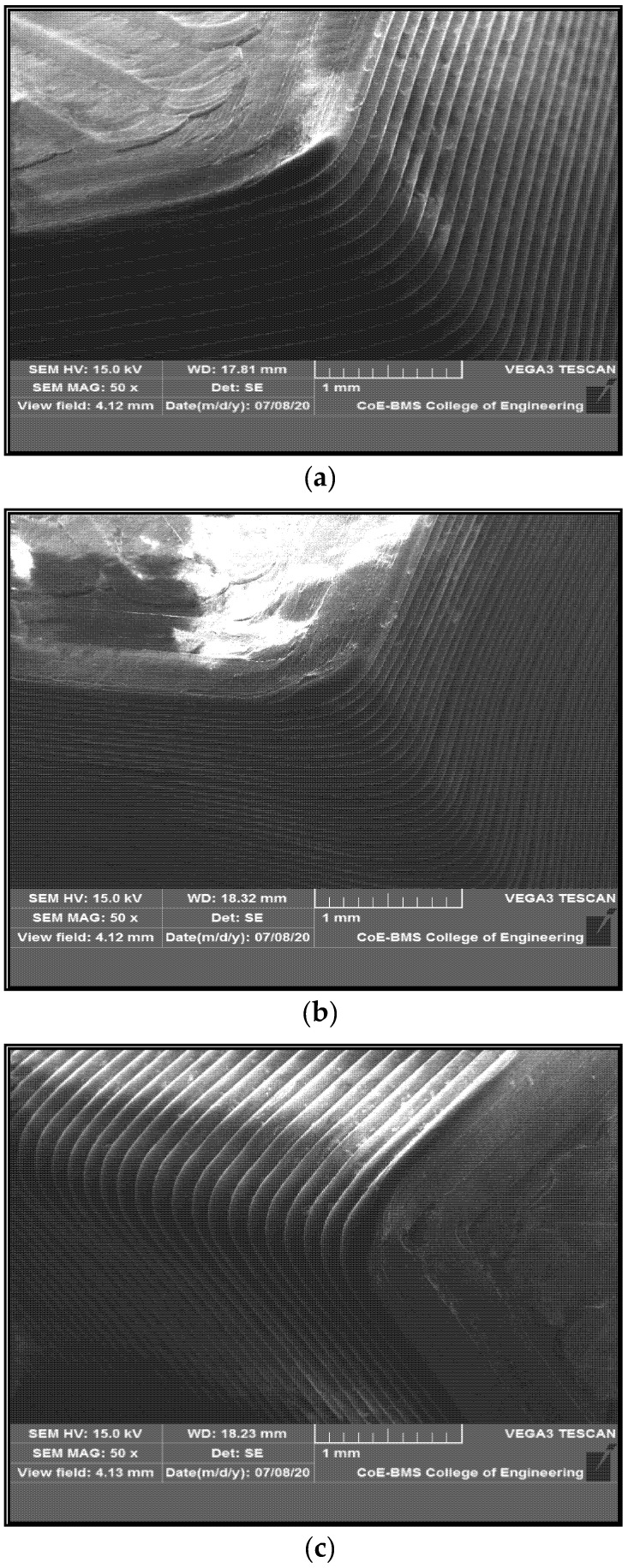
Isometric view of FDM-printed parts of (**a**) PC-ABS, (**b**) PC-ABS + 0.4 wt % graphene, (**c**) PC-ABS + 0.8 wt % graphene.

Figure 14 shows the SEM images of FDM parts in the isometric view in a layer-wise manner, indicating the proper alignment and dimensional stability for both PC-ABS and composites with the addition of graphene.

### 3.5. Mechanical Analysis

#### 3.5.1. Tensile Strength

**Figure 15:**
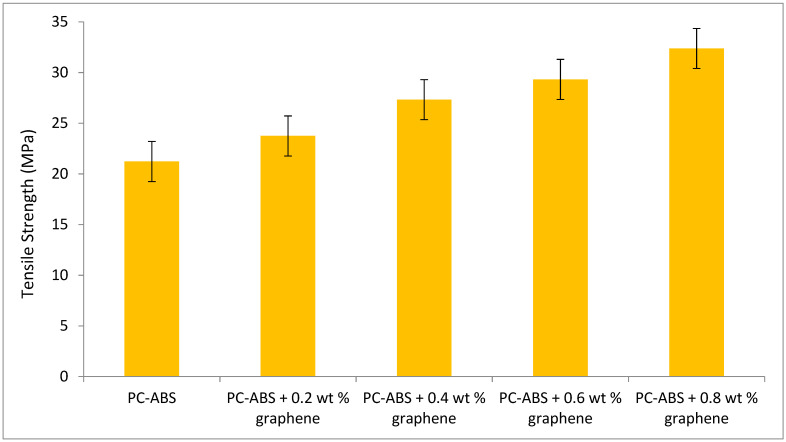
Variation of tensile strength with increase in graphene content.

ASTM D638 testing method was followed to determine the ultimate tensile strength of the fabricated part. The graph in Figure 15 indicates the increase of the ultimate tensile strength (UTS) with the addition of graphene from 0.2 wt % to 0.8 wt %. Tensile strength increased by 9% with the addition of 0.2 wt % of graphene when compared to pure PC-ABS. The increment in tensile strength was increased by 12% with the addition of 0.4 wt % of graphene. The increment in the value is in linear trend with the addition of filler content. With a maximum addition of 0.8 wt % graphene in this experiment, the tensile strength increased by 57% compared to pure PC-ABS.

The increased tensile strength with graphene addition is attributed to the massive intermolecular linkages and reinforcement alignment [32]. This property has classified graphene as a compatibilizer because it forces significant interaction with the polymer matrix, resulting in linkages. Graphene’s self-lubrication property is improved by changing the primary packing arrangements, which in turn alters the binding force [33]. The addition of graphene to PC-ABS improves the interfacial bonding network, thereby improving thermal stability [34]. Hsu et al. conducted a mechanical study on the effect of graphene nanosheets on polypropylene. The addition of 0.2 wt % graphene-enhanced polypropylene increased the tensile strength to 29.54 MPa by 8.24% when compared to pure polypropylene. The wide dispersion of graphene in the polypropylene matrix has resulted in an enhanced improvement in tensile strength. However, the addition of graphene nanosheets greater than a certain weight % would result in agglomeration, wrapping of nanosheets, and poor dispersion, resulting in a decrease in tensile properties [35]. The increase in tensile strength depends on loading direction and reinforcement alignment. The highest tensile strength is achievable when the load direction and reinforcement direction are similar; a study carried out by Ahn et al. [36] suggested the effect of varying process parameters on the anisotropy of the material properties in FDM, and emphasized that for maximum tensile loads, the load direction should align with the reinforcement orientation, which is similar to the observations made in our experimental study.

**Figure 16:**
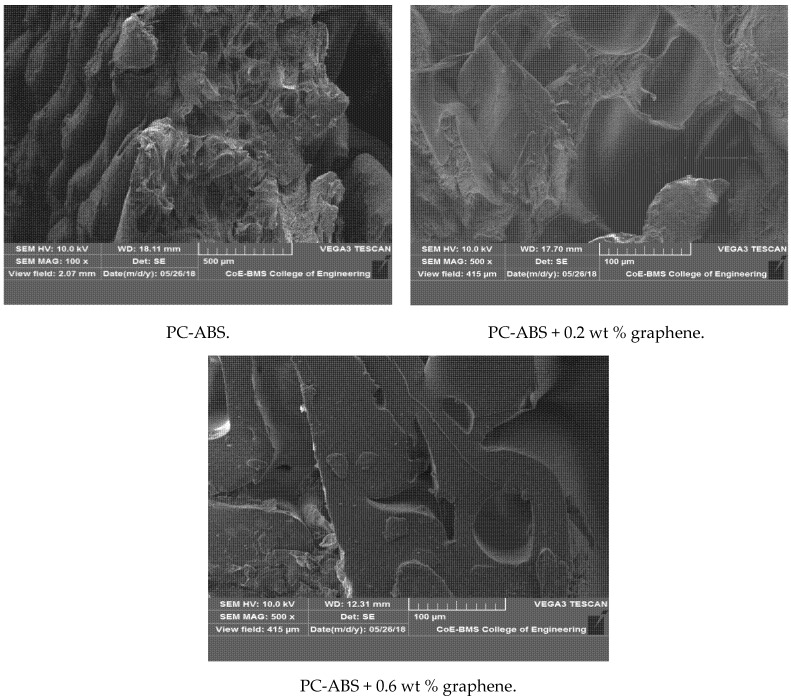
SEM of fractured tensile specimens of PC-ABS, PC-ABS + 0.2 wt % graphene, and PC-ABS + 0.6 wt % graphene.

**Figure 17:**
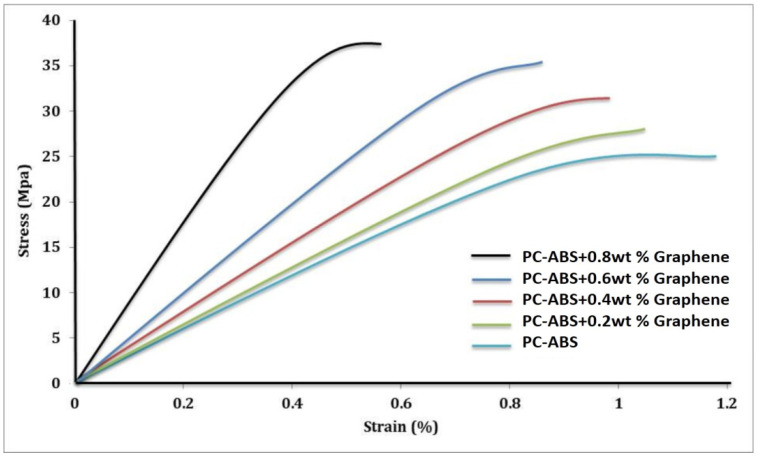
Stress–strain graph of PC-ABS and its composites.

Figure 16 depicts the SEM of the fractured specimens obtained through tensile testing. The samples were printed using the FDM technique, demonstrating the reduction in voids with the addition of graphene. The physical properties of the blended nanocomposite are improved by proper blending and dispersion. Tenikalpet et al. [37] participated in a similar study, conducting a tensile strength comparison between conventionally manufactured specimens and specimens developed using fused deposition modelling. Glass fiber with an average length of 3.5 mm was reinforced in various proportions with ABS matrix. Tensile strength was found to be higher in conventionally manufactured specimens than in specimens developed using the fused deposition modelling process. However, as the wt percent of filler content increased, so did the tensile strength. It was also discovered that increasing the filler content increased the void in the beads while decreasing the voids between the beads. The mechanical properties of the developed nanocomposite are shown in Table 2. The incremental addition of graphene results in an increase in modulus. When compared to virgin PC-ABS, PC-ABS + 0.8 wt percent graphene has a 59.4 percent increase. Zare et al. [38] demonstrated that proper stress transfer between the matrix and reinforcement can improve the mechanical performance of polymer nanocomposite; this can be accomplished with adequate adhesion. The analysis of Young’s modulus reveals an increase in thermal and mechanical properties. This is attributed to the fine level of interaction in the interphase and between PC-ABS and graphene. The increase in yield strength is observed with the incremental addition of graphene. A 34.32% increase in yield strength has been reported for PC-ABS + 0.8 wt % when compared to pure PC-ABS. However, percentage elongation has been reduced with every increment of graphene to the PC-ABS matrix. All the graphene-filled composites exhibited relatively brittle behavior. Five samples have been tested for each composition, and the modulus of elasticity was computed in the range of 0 to 20 MPa. The Young’s modulus was obtained from the slope of a linearly fitted line with the data at strains smaller than 0.2%. Figure 17 shows the stress–strain graph of all the specimens.

Seyeon et al. developed a polymer nanocomposite using ABS as a matrix and iron and copper as filler material. The tensile strength analysis showed that the specimens printed with 3D printing technology would have better tensile properties with increased fill density. An impressive result was revealed such that as the increment in metal filler increases above a specific value, the decrement in tensile strength is observed [39].

#### 3.5.2. Impact Strength

**Figure 18:**
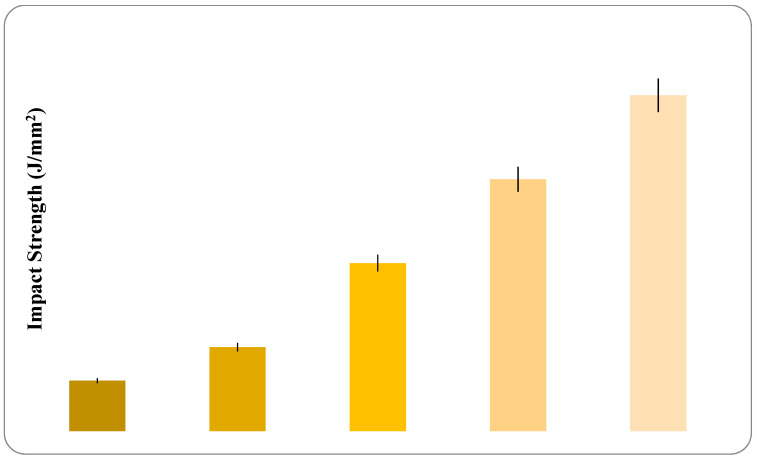
Variation in impact strength with increase in graphene content.

The impact strength of pure PC-ABS had a value of nearly 1 J, which increased with the incremental addition of graphene reinforcement; 0.4 wt % addition of graphene gave an incremental value of nearly 4 J, an increase of 75% with impact resistance. The highest value of 87% was observed for the addition of 0.8 wt % of graphene. Suarez et al. have stated that the addition of PC to ABS increased the developed specimens’ impact strength [40]. An increase in resistance to breaking energy with the addition of graphene is seen in Figure 18. The incremental breaking resistance energy with the addition of graphene is increasing for every incremental addition of graphene in an overall manner. However, the results show the reduction in breaking energy percent with increased graphene content: the incremental percent from 0.2 wt % to 0.4 wt % is 100%, from 0.4 wt % to 0.6 wt % 50% and so on.

**Figure 19:**
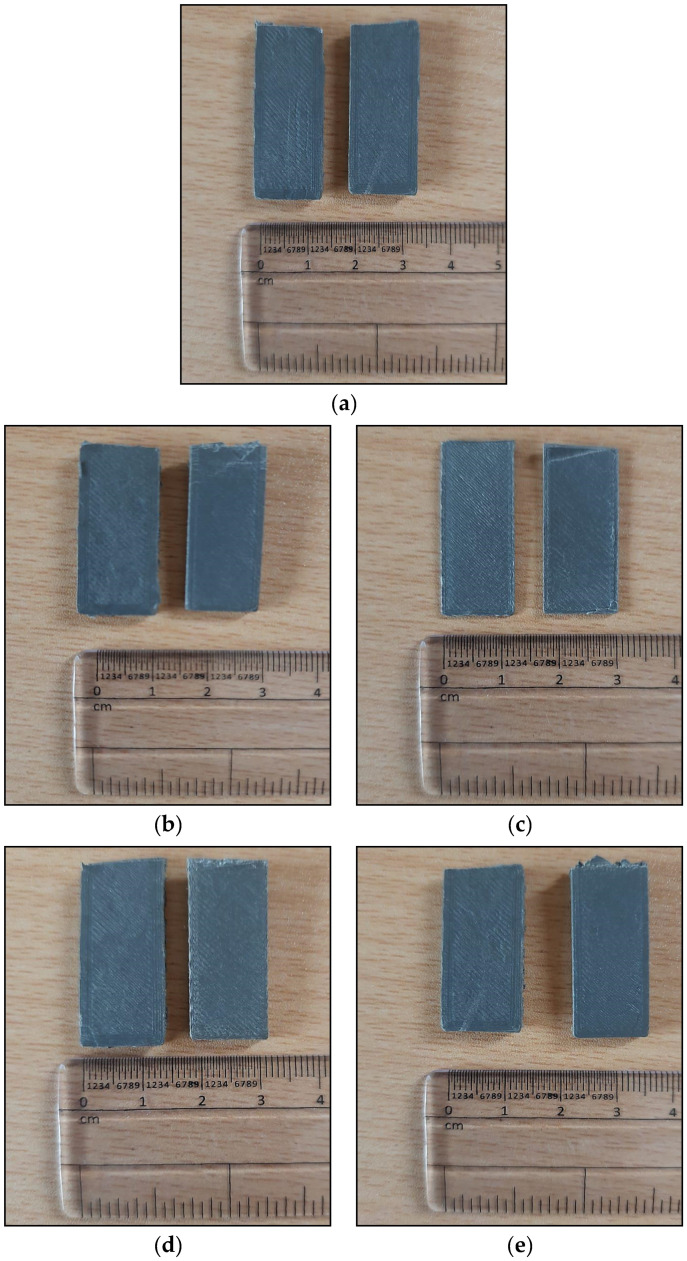
Photographs of fractured specimens after impact test: (**a**) PC-ABS; (**b**) PC-ABS + 0.2 wt % graphene; (**c**) PC-ABS + 0.4 wt % graphene; (**d**) PC-ABS + 0.6 wt % graphene; (**e**) PC-ABS + 0.8 wt % graphene.

The incorporation of graphene into the matrix tends to inhibit deformation and ductile mobility of polymer molecules while also absorbing energy during crack propagation. Fewer flaws in composites result from fewer void spaces, which improve composite impact strength. Figure 19 depicts a photograph of fractured specimens of PC-ABS and composites with varying graphene percentages. The photograph shows that all of the samples have completely broken and have the same fracture characteristics, despite the fact that the fracture location varies slightly. Due to the presence of a little residual material in the samples, the appearance of the composite specimens differs marginally from PC-ABS. Because of the inhomogeneous and anisotropic structure of the composite materials, combined with the material’s hardening process, the shape of the resulting fracture indicates that the material is slightly brittle.

## 4. Conclusions

The following conclusion has been derived from the experimental study carried out in determining the mechanical properties of polymer nanocomposites developed by FDM:

Fixed filament of required diameter was successfully carried out for PC-ABS + graphene filament by compounding and twin-screw extrusion.

The PC-ABS and composite specimens have been tested perpendicular to printing, as this direction offers the highest mechanical properties in the 3D printing process.

Microstructure studies indicate proper dispersion of graphene in the PC-ABS matrix and elemental mapping suggests no formation of aggregates.

Reduction in surface roughness was noted for every incremental addition of graphene to PC-ABS. A 0.2 wt % addition of graphene showed a decrement of 25.8% in comparison to PC-ABS. The highest decrement was seen for the 0.8 wt % addition of graphene reinforcement that amounted to 40% compared to PC-ABS.

The increment in tensile strength is in linear trend with the addition of graphene. With a maximum addition of 0.8 wt % graphene in this experiment, the tensile strength increased by 57% compared to pure PC-ABS. A 34.32% increase in yield strength has been noted for PC-ABS + 0.8 wt % when compared to pure PC-ABS. Percentage elongation was reduced with every increment of graphene added to the PC-ABS matrix.

An increase in resistance to breaking energy with the addition of graphene has been observed.

Graphene nanosheets addition has significantly improved the physical and mechanical properties of FDM parts.

## Figures and Tables

**Table 1:** Surface roughness (Ra) values of FDM parts.

Specimen	Average Surface Roughness (Ra) in X Direction	Average Surface Roughness (Ra) in Y Direction	Average Surface Roughness (Ra) in Z Direction
PC-ABS	0.703	0.832	0.799
PC-ABS + 0.2 wt % Graphene	0.512	0.795	0.673
PC-ABS + 0.4 wt % Graphene	0.486	0.644	0.578
PC-ABS + 0.6 wt % Graphene	0.425	0.498	0.512
PC-ABS + 0.8 wt % Graphene	0.416	0.481	0.483

**Table 2:** Modulus, yield strength, and % elongation of PC-ABS and its composites.

Material	Modulus (E, GPa)	Yield Strength (MPa)	% Elongation
PC-ABS	2.53 + 0.15	19.83 + 2.5	1.12 + 0.3
PC-ABS + 0.2 wt % Graphene	2.80 + 0.2	21.32 + 3.1	1.08 + 0.2
PC-ABS + 0.4 wt % Graphene	3.23 + 0.2	24.51 + 3.5	1.02 + 0.4
PC-ABS + 0.6 wt % Graphene	3.81 + 0.22	25.74 + 2.9	0.94 + 0.41
PC-ABS + 0.8 wt % Graphene	4.03 + 0.3	30.14 + 3.4	0.54 + 0.32

## Data Availability

The data presented in this study are available on request from the corresponding author.
